# Prevalence and factors associated with suicidal ideation and attempts among mentally ill patients in the psychiatry OPD at St. Paul's Hospital Millennium Medical College, Addis Ababa, Ethiopia

**DOI:** 10.3389/frcha.2024.1342257

**Published:** 2024-04-10

**Authors:** Awol Dawud Mohammed, Takele Menna, Mohammed Ayalew, Tesfalem Teshome, Mikiyas Alayu, Neamin Tesfay

**Affiliations:** ^1^ Public Health Emergency Management, Ethiopia Public Health Institute, Addis Ababa, Ethiopia; ^2^ Department of Epidemiology, School of Public Health, St Paul's Hospital Millennium Medical College, Addis Ababa, Ethiopia; ^3^ Department of Psychiatry, Hawasa University, Hawasa, Ethiopia

**Keywords:** suicidal ideation, prevalence, mentally ill, suicidal attempts, risk factor

## Abstract

**Background:**

Every year, more than 800,000 people die due to suicide (one person every 40 s), ranking as the second leading cause of death next to traffic accidents in individuals aged 15–29 years. The objective of this study was to assess the prevalence and factors associated with suicidal ideation and attempts among mentally ill patients.

**Method:**

An institutional-based, cross-sectional study was conducted between December 2019 and January 2020. Data were collected using a convenient sampling technique until the required sample size was achieved. The total sample size was 385. The Suicidal Behavior Questionnaire-Revised (SBQ-R), Oslo Social Support Scale, and self-prepared to assess suicide were used. Data collection was carried out by psychiatry professionals through face-to face interviews. Data were analyzed, and bivariate and multivariable logistic regression analyses were conducted using Statistical Package for the Social Sciences (SPSS) version 23.

**Result:**

A total of 385 patients participated in the study. Of these, 216 (56%) were men. The prevalence of suicide ideation was 255 (66.2%). The odds of suicidal ideation and attempt was almost three times higher [adjusted odds ratio (AOR) = 2.79, 95% confidence interval (CI) 1.11–6.98]; among patients who felt hopeless, the OR was around two times higher (AOR = 2.44, 95% CI 1.17–5.08); among patients who had a family history of suicide, the OR was almost three times higher (AOR = 2.56, 95% CI 1.00–6.53); among patients who stopped their medication, the OR was approximately two times higher (AOR = 2.25, 95% CI 1.14–4.46); the OR of suicidal ideation and attempts was almost six and four times higher (AOR = 5.86, 95% CI 1.30–26.41; and AOR = 3.61, 95% CI 1.01–12.88) among patients who were educated to primary and secondary level, respectively.

**Conclusion:**

In this study, carried out in Ethiopia, suicidal ideation and attempts were more common among men than women. The prevalence of suicidal ideation and attempts showed a significant public health issue among hospital-attending and chronic mentally ill adolescents, which requires a large emphasis. We recommend that suicide prevention needs to involve non-governmental and non-profit organizations, universities, and civil society at different levels.

## Background

Suicide is a serious, preventable public health problem that results in social, emotional, and economic consequences for family, friends, and colleagues (1). Because suicide remains a sensitive issue, it is very likely that it is underreported due to stigma, criminalization, and weak surveillance systems. It demands our attention and action even though its prevention and control are difficult (2).

Several environmental, psychosocial, and behavioral factors have been found to be associated with suicidal ideation, suicidal attempts, and suicide. Suicidal behaviors often coexist with other health risk behaviors, such as aggressive behavior, smoking, and experiences of sexual intercourse (3, 4). Suicide ideation and attempted suicide were also related to many severe mental health problems, such as anxiety, schizophrenia, bipolar disorder, mood disorder, depression, and others (5).

The method of the suicide attempt influences the morbidity and completion rates, independent of the severity of the intent to die at the time of suicidal behavior. The most common methods of attempting suicide are hanging, poisoning, slashing, and shooting (6, 7).

The burden of suicide constitutes a serious public health issue worldwide, and mental health professionals need to increase their awareness of the warning signs for suicide. Suicide warning signs are associated with acute factors that inform clinicians about observable signs and expressed emotions, and are important for saving lives by early detection and intervention for those at risk (8).

Every year, more than 800,000 people die due to suicide (one person every 40 s), ranking it the second leading cause of death next to traffic accidents in individuals aged 15–29 years. Among nearly 250 causes of death, suicide was the 14th leading cause of global mortality (9). In addition, it is also predicted that, by 2020, the rate of death due to suicide will be increased to one every 20 s (10). Suicide accounts for 50% and 71% of all violent deaths globally in men and women, respectively.

Internationally, suicide rates range from highs of more than 25 per 100,000 individuals in Scandinavia, Switzerland, Germany, Austria, the eastern European countries, and Japan to less than 10 per 100,000 in Spain, Italy, Ireland, Egypt, and the Netherlands (11). Rates of suicide among adolescents and young adults have increased considerably in recent decades (12).

In sub-Saharan Africa, death from suicide is estimated to be 34,000 per year (13). The prevalence of suicide ideation was estimated to be 7% in Tanzania (3), 6.2% in Seychelles (14), 31.3% in Zambia (15), 23.3% in Benin (5), and 21.6% in Uganda. This even occurs even though some mental health problems may arise from infectious diseases (16). However, in Ethiopia, there was no adequate research on the magnitude or burden of suicidal ideation and attempts. The aim of the present study was to determine the prevalence of suicidal ideation and attempts and the factors associated with suicidal ideation and attempts among mentally ill patients.

## Method

### Study area

Saint Paul's Hospital Millennium Medical College (SPHMMC) was established at the center of the country, in Addis Ababa, through a decree of the Council of Ministers in 2010, although the medical school was opened in 2007. The hospital was established in 1968 by the late Emperor Haile Selassie. It is governed by a board under the Federal Ministry of Health. The SPHMMC has many specialist programs and has many departments, including a psychiatry department. The psychiatry department started approximately 40 years earlier, i.e., in the 1975 Ethiopian calendar.

### Study design and period

An institutional-based, cross-sectional study was conducted between December 2019 and January 2020.

### Sample size determination

The sample size was determined using a single population proportion formula as follows:

$$\;n\;=\;\frac{{\left(Z_{a/2}\right)}^{2}P(1-P)}{d^{2}}$$

where *P* = the prevalence 64.8% (0.648) from the study by Mekonnen and Kebede (17), and the absolute precision or tolerable margin of error (*d*) = 5% (0.05). *Z*_*α*/2_ = *Z* value at (*α* = 0.05) = 1.96 corresponding to the 95% confidence interval (CI). After adjusting for a 10% contingency for non-response rates, a total of 385 study populations were involved in the study.

### Sampling techniques and procedures

All patients presenting at the SPHMMC Psychiatry Patient Department (outpatient department, OPD) between December 2019 and January 2020 were included in this study using a consecutive sampling technique until the required sample size was achieved. Both new and follow-up patients were included in the study.

### Data collection procedures and instruments

Data were collected using an interviewer-administered structured questionnaire. The questionnaire included sociodemographic characteristics, patient clinical characteristics, social support of the patient, environmental risk factors, methods used for suicide attempts, and items related to patients’ suicidal behavior, i.e., ideation and attempt. A pretest was carried out with 5% of the sample size at St. Peter Specialized Hospital among patients with mental illness before the actual study was conducted and the data collection tool was revised based on the findings of the pretest. Data collectors were 10 trained psychiatric nurses; 2 senior mental health experts were assigned as supervisors to the data collectors. The patients were diagnosed by psychiatrists, psychiatry residents, and senior or mental health professionals. Therefore, the diagnosis and some clinical factors were reviewed from each patient's chart.

Items used to assess suicidal ideation and attempt were used, adapted from Suicidal Behavior Questionnaire-Revised (SBQ-R) (18). The items included in the questionnaire were as follows: item 1 taps into lifetime suicidal ideation and attempt; item 2 assesses the frequency of suicidal ideation over the past 12 months; and item 3 taps into the threat of suicidal behavior. The level of social support among patients with mental illness was assessed using the three-item Oslo Social Support Scale (OSSS) and the scores were in the range of 3–14 (19).

### Data processing and analysis

Data were checked for completeness and consistency, and then coded and entered into Epi info version 7.2.1.0. They were then exported to Statistical Package for the Social Sciences (SPSS) version 23 for analysis. Descriptive analyses of ratios, proportions, and rates were computed. Bivariate and multivariate logistic regression analyses were also conducted. Variables with a *P*-value <0.2 in the bivariate analysis were fitted to multivariable logistic regression. Those variables with a *P*-value <0.05 in the multivariable analysis were considered to be significant.

## Results

### Sociodemographic characteristics

A total of 385 participants (age range = 18–85 years) were enrolled in the study, with a response rate of 100%. Of the total participants, 216 (56%) were men. Of the participants, 197 (51%) were single, 232 (48.9%) were illiterate, 140 (36.4%) had a level of education from grade 9 to 12, and 77 (18.4%) were private/non-governmental organization (NGO) employees (Table 1).

**Table 1:** Sociodemographic characteristics of study participant patients with mental illness were attending the Psychiatric Department of SPHMMC in Addis Ababa.

No.	Variables	Category	Frequency	Percentage
1	Age (years)	28–37	133	34
		18–27	95	25
		38–47	79	21
		≥48	78	20
2	Sex	Male	216	56
		Female	169	44
3	Marital status	Single	197	51
		Married	126	33
		Divorced	39	10
		Widowed	23	6
4	Education	9th–12th grade	140	37
		1st–8th grade	109	28
		College and above	105	27
		Illiterate	31	8
5	Occupation	Private/NGO Employee	71	18
		No job	60	16
		Gov’t employee	59	15
		Housewife	52	14
		Merchant	42	11
		Student	42	11
		Daily laborer	31	8
		Farmer	8	2
		Other	20	5
6	Income (Birr)	<1,000	195	51
		1,000–2,000	56	14
		2,000–4,000	61	16
		≥5,000	73	19
7	Residence	Rural	23	6
		Urban	362	94
8	Social support	Poor	168	44
		Moderate	136	35
		Strong	81	21

### Clinical characteristics of participants

In total, 297 (77%) participants were diagnosed as mentally ill before presenting at SPHMMC. As to the duration of being psychiatric patients, among the study participants, 195 (51%) reported that they experienced mental illnesses for more than 5 years before coming to SPHMMC (Table 2).

**Table 2:** Clinical characteristics of participants visiting psychiatric OPD of SPHMMC, Addis Ababa, 2020.

No.	Variables	Category	Frequency	Percentage
1	Having mental illness before coming here?	Yes	297	77
		No	88	23
		>60 months	195	51
		13–60 months	82	21
		≤12 months	20	5
3	Having chronic diseases	Yes	51	13
		No	334	87
4	Family history of mental illness	Yes	183	48
		No	202	52
5	Family history of suicide	Yes	79	21
		No	306	79
6	Stopping of medication	Yes	156	41
		No	229	59
7	Episode of the illness	No episode	151	39
		Single episode	108	28
		2–4 episodes	92	24
		5 and above	19	5
		Continuous	15	4
8	Substance use	Yes	89	23
		No	296	77
9	Substance	Khat	72	19
		Alcohol	70	18
		Cigarette	70	18
		Hashish	9	2
		Ganja	8	2
		Cocaine	2	1
		Cannabis	1	0.50

Of the participants with suicidal ideation and attempts, 71 (34.4%) were diagnosed with major depression disorder (MDD) and 53 (25.6%) were diagnosed with schizophrenia (Table 3).

**Table 3:** Mental illnesses diagnosed among the study participants and those who reported in both suicidal ideation and attempts during the study period, SPHMMC, 2020.

Sno.	Variable (Diagnosis)	Suicidal ideation and attempts	
		Frequency	Percentage
1	MDD	71	34.4
2	Schizophrenia	53	25.6
3	Bipolar	28	13.5
4	Substance use disorder	21	10
5	Generalized anxiety disorder	14	7
6	Seizure disorder	7	3.4
7	Somatic disorder	4	2
8	Other mental illness^a^	9	4

^a^ Other illness: epilepsy 3, panic 2, hypersomnia 1, social phobia 1, post-prenatal psychosis 1, and post-traumatic 1.

### Prevalence of suicide ideation and attempts

Of the 385 respondents, 255 (66.2%) [men = 143 (37%), women = 112 (29%)] had suicidal ideation and 210 (55%) attempted suicide [men = 115 (30%), women = 95 (25%)]. Of the study participants, 207 (54%) had thought of suicide and attempt. Of them, 19 (5%) were told by someone that they were going to commit suicide or they might have to do it in their lifetime (Table 4).

**Table 4:** Frequency distribution of suicide ideation and attempt among mental Ill patients in SPHMMC, Addis Ababa, Ethiopia, 2020 (*n* = 385).

Variable	Category	Frequency	Percentage	Male	Percentage	Female	Percentage
Have you ever thought about killing yourself?	Yes	255	66	143	37	112	29
	No	130	34	73	19	57	15
Have you ever attempted to kill your self	Yes	210	55	115	30	95	25
	No	175	45	101	26	74	19
Suicidal ideation and attempts?	Yes	207	54	113	29	94	25
	No	178	46	103	27	75	19
Have you ever told someone commit suicide	Yes	19	5	12	3	7	2
	No	239	62	133	35	106	28
Suicidal ideation or attempts?	Yes	258	67	145	38	113	29
	No	127	33	71	18	56	15

**Figure 1:**
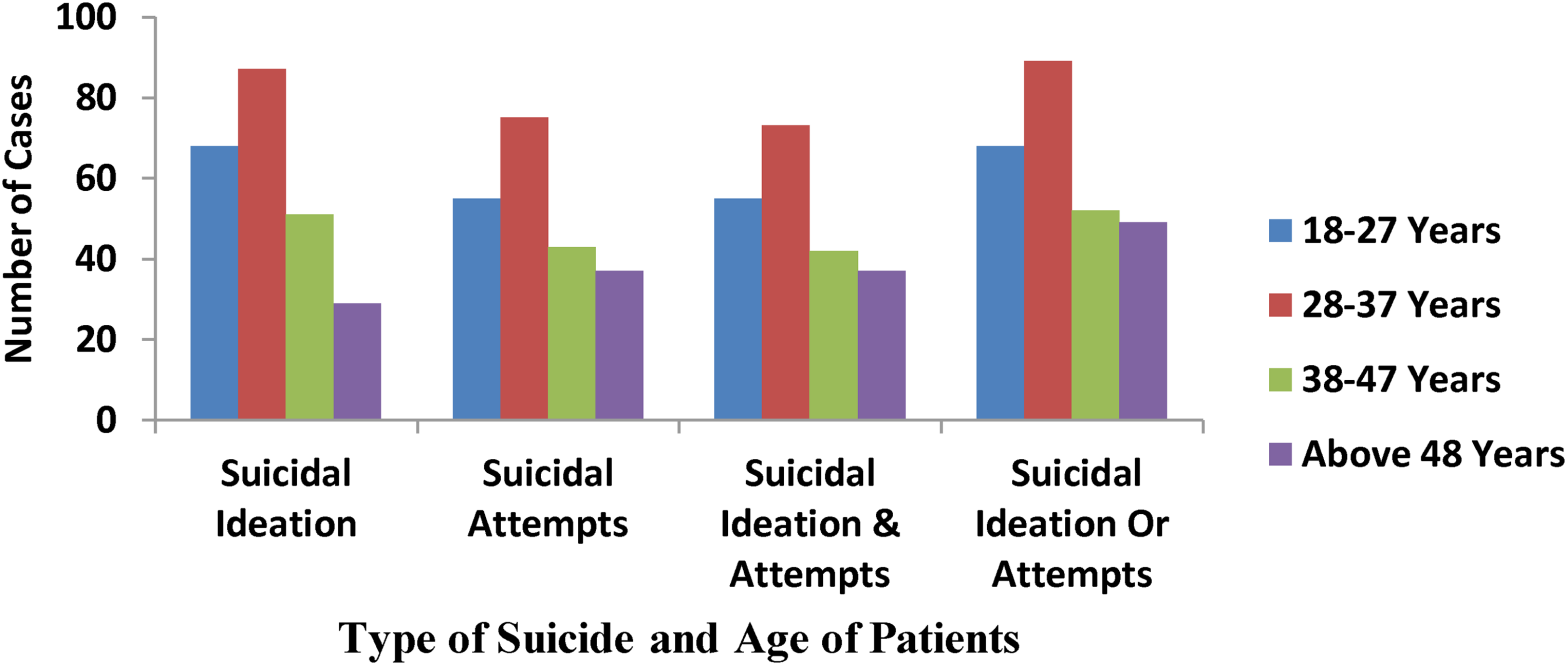
Distribution of suicide ideation and attempt by age among mental Ill patients of psychiatry OPD of SPHMMC, Addis Ababa, Ethiopia, 2020 (*N* = 385).

Among all participants, the age range of 28–37 years was the most affected age group (see Figure 1).

**Figure 2:**
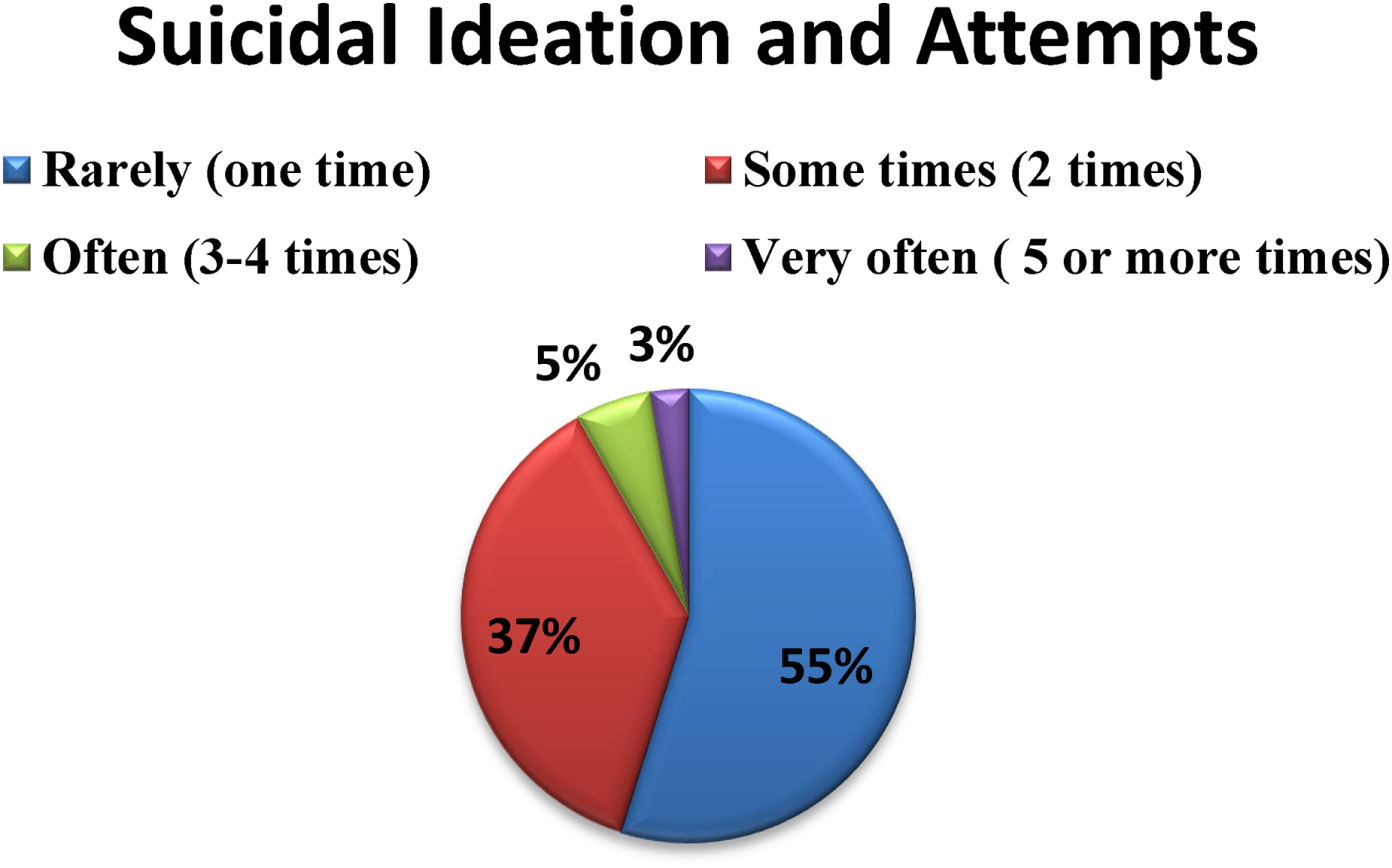
Percentage distribution of frequency of suicide ideation and attempt among mental Ill patients in SPHMMC, Addis Ababa, Ethiopia, 2020 (*n* = 385).

Of the participants, 142 (55%) rarely had suicidal ideation and attempts (one time only) and 7 (3%) had five or more suicidal thoughts and attempts (Figure 2).

### Methods of suicidal ideation and attempts

Of the participants, 98 (47%) thought of attempting suicide using drug overdose/poisoning and 92 (44%) of them thought of attempting suicide by hanging themselves. The smallest proportion of participants (*n *=* *5, 2%) thought of attempting suicide using a gun.

**Figure 3:**
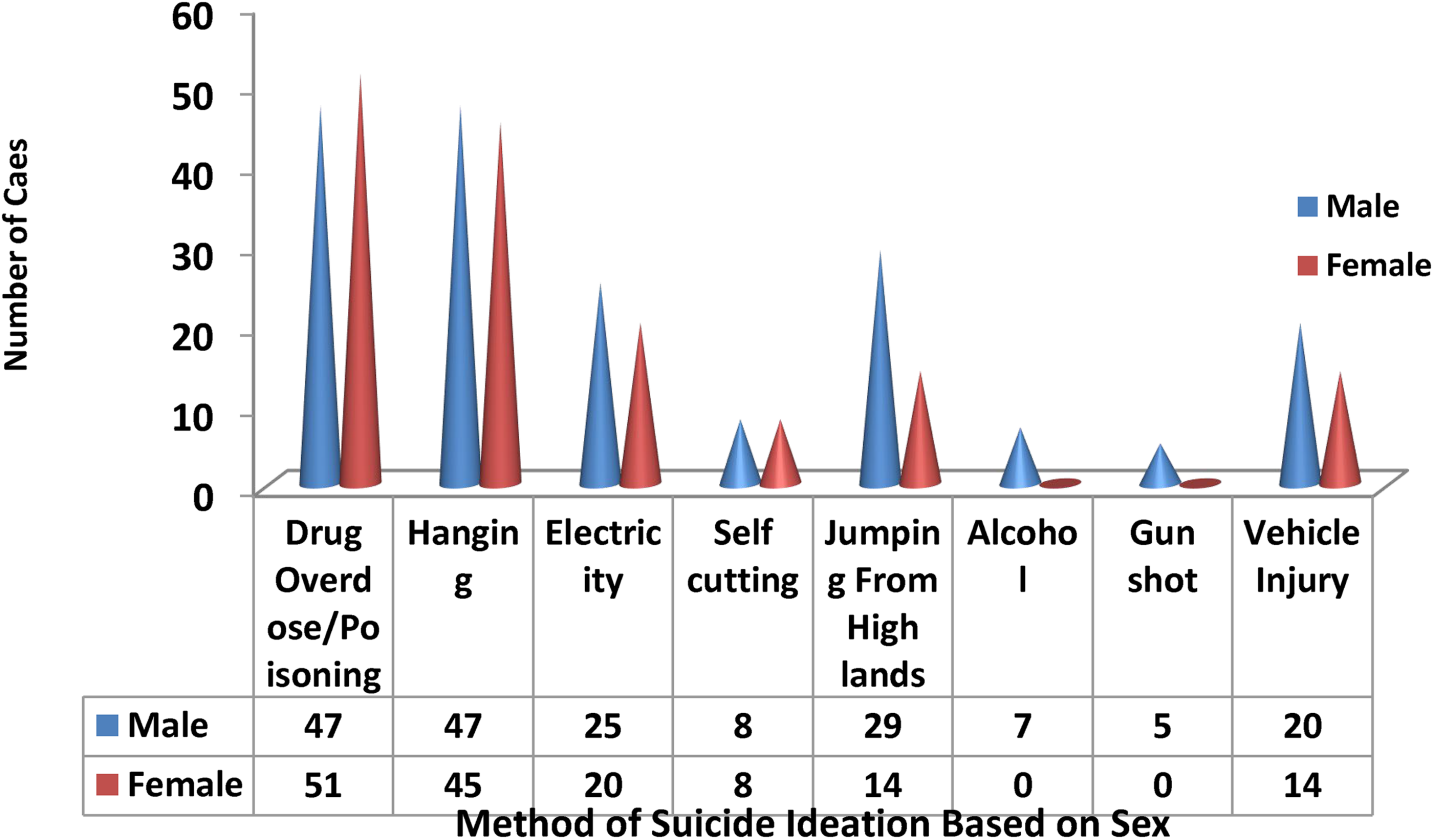
Frequency distribution of methods of suicidal ideation and attempts at sex among mentally Ill patients in SPHMMC, Addis Ababa, Ethiopia, 2020.

From all methods listed in the questionnaire and the participants applied to them and thought to kill themselves based on sex drug dose/poisoning were the highest one female (51) and male (47) (Figure 3).

### Factors associated with suicidal ideation and attempt

In the bivariate analysis, the following information was collected: age, marital status, occupation, education, religion, monthly income, residence of the participant, duration of mental illness, family history of mental illness, family history of suicide, stopped mental illness medication, Oslo Social Support Scale, recurrence/relapse of mental illness, current psychiatric diagnosis, feelings of hopelessness, having mental health issues, and death in family (Table 5).

**Table 5:** Bivariate logistic regression results of suicide ideation and attempt among mental Ill patients in SPHMMC, Addis Ababa, Ethiopia, 2020 (*n* = 385).

Variables	Category	Suicide ideation and attempt		Crude odd ratio (95% CI)	*P*-value
		Yes	No		
Age (years)	18–27	55	40	1.348 (0.770–2.361)	0.296
	28–37	73	60	1.524 (0.834–2.784)	0.171
	38–47	42	37	1.258 (0.672–2.352)	0.473
	≥48	37	41	1	
Marital status	Single	111	86	0.830 (0.343–2.007)	0.679
	Divorced	14	25	0.360 (0.124–1.042)	0.060
	Married	68	58	0.754 (0.304–1.868)	0.541
	Widowed	14	9	1	
Educational status	Illiterate	12	19	0.669 (0.295–1.515)	0.335
	1–8th grade	60	49	1.297 (0.757–2.219)	0.344
	9–12th grade	84	56	1.588 (0.953–2.267)	0.076
	≥College	51	54	1	
Occupation	Farmer	5	4	1.500 (0.288–7.807)	0.630
	Housewife	32	21	2.400 (0.836–6.891)	0.104
	Merchant	22	21	1.650 (0.560–4.860)	0.364
	Gov’t employee	31	28	1.661 (0.593–4.653)	0.335
	Private/NGO employee	37	38	1.378 (0.503–3.779)	0.533
	Student	25	16	2.437 (0.820–7.250)	0.109
	Daily labor	22	20	1.821 (0.582–5.698)	0.303
	Jobless	33	29	1.833 (0.655–5.131)	0.248
	Others	5	5	1	
Income (Birr)	<1,000	113	82	1	
	10,001–2,000	28	28	0.726 (0.400–1.317)	0.292
	2,001–4,000	29	32	0.658 (0.369–1.171)	0.155
	>4,000	37	36	0.746 (0.435–1.280)	0.287
Family history of mental illness	Yes	119	64	2.40 (1.59–3.63)	≤0.001
	No	88	114	1	
Family history of suicide	Yes	59	20	3.14 (1.80–5.48)	≤0.001
	No	148	158	1	
On medication for mental illness	Yes	183	147	1	
	No	24	31	0.62 (0.35–1.11)	0.106
Stop medication	Yes	108	48	2.95 (1.924–4.537)	≤0.001
	No	99	130	1	
Current psychiatric diagnosis	Schizophrenia	52	50	1	
	MDD	97	40	1.61 (0.93–2.76)	0.090
	Bipolar disorder	28	18	1.49 (0.73–3.03)	0.265
	Other psychotic disorder	3	14	0.20 (0.05–0.76)	0.018
	Anxiety disorders	18	17	1.02 (0.47–2.19)	0.963
	Substance use disorder	21	18	1.12 (0.53–2.35)	0.761
	Somatic disorder	4	13	0.29 (0.09–0.96)	0.044
	Other disorder	14	8	1.68 (0.65–4.35)	0.284
Social support	Poor	112	56	2.27 (1.60–4.77)	≤0.001
	Moderate	61	75	1.12 (0.64–1.96)	0.679
	Strong	34	47	1	
Feeling of hopeless (*N* = 258)	Yes	153	29	2.14 (1.13–4.05)	0.018
	No	54	22	1	
Duration of illness (months) (*N* = 297)	≤12	10	10	0.95 (0.37–2.38)	0.913
	13–60	51	31	1.56 (0.92,2.64)	0.097
	≥61	100	95	1	
Recurrences/relapse of mental illness	Continuous	11	4	5.39 (1.63–17.77)	0.006
	Single	61	47	2.54 (1.53–4.23)	≤0.001
	2–4	69	23	5.88 (3.29–10.50)	≤0.001
	>5	15	4	7.35 (2.32–23.30)	0.001
	No relapse	51	100	1	
Family death	Yes	48	7	1.89 (0.80–3.04)	0.183
	No	159	44	1	

The odds of suicidal ideation and attempt were lower among divorced people [adjusted odds ratio (AOR) = 0.31, 95% CI 0.11–0.85, *p*-value = 0.022] compared to single people; individuals with a primary and secondary education had a more likely risk of suicide ideation and attempts than those educated to college level and above (AOR = 5.86, 95% CI 1.30–26.41; and AOR = 3.61, 95% CI 1.01–12.88), respectively (Table 6). Individuals who stopped their mental illness medication had a higher risk of suicide ideation and attempt than those continuing with their medication (AOR = 2.56, 95% CI 1.00–6.53), and individuals with a family history of suicide had a more likely risk of suicide ideation and attempts than those who do not have a family history of suicide or higher (AOR = 2.44, 95% CI 1.17–5.08) (Table 1).

**Table 6:** Multivariate logistic regression on prevalence and associated factors of suicidal ideation and attempts among mentally Ill patients in psychiatry OPD at SPHMMC, in Addis Ababa, Ethiopia, 2020 (*n* = 385).

Variables	Category	Suicide ideation and attempt		AOR (95% CI)	*P*-value
		Yes	No		
Marital status	Single	111	86	1	
	Divorced	14	25	0.31 (0.11–0.85)	0.022
	Married	68	58	0.90 (0.45–1.81)	0.771
	Widowed	14	9	1.61 (0.39–6.58)	0.506
Educational status	Illiterate	12	19	7.71 (0.84,70.44)	0.070
	1–8th grade	60	49	5.86 (1.30–26.41)	0.021
	9–12th grade	84	56	3.61 (1.01–12.88)	0.048
	≥College	51	54	1	
Social support	Poor	112	56	2.25 (1.14–4.46)	0.020
	Moderate	61	75	0.66 (0.33–1.35)	0.667
	Strong	34	47	1	
Family history of mental illness	Yes	119	64	1.94 (1.12–3.36)	0.017
	No	88	114	1	
Family history of suicide	Yes	59	20	2.44 (1.17–5.08)	0.017
	No	148	158	1	
Stop psychiatric medication	Yes	108	48	2.56 (1.00–6.53)	0.049
	No	99	130	1	
Current psychiatric diagnosis	Schizophrenia	52	50	1	
	MDD	97	40	1.70 (0.85–3.42)	0.135
	Bipolar D/os	28	18	1.49 (0.62–3.60)	0.377
	Other psychotic D/os	3	14	0.13 (0.03–0.61)	0.010
	Anxiety disorders	18	17	1.68 (0.66–4.28)	0.278
	SUD	21	18	1.39 (0.45–4.34)	0.561
	Somatic D/os	4	13	0.47 (0.10–2.13)	0.325
	Other D/os	14	8	2.29 (0.72–7.28)	0.159
Felt hopeless	Yes	153	29	2.79 (1.11–6.98)	0.028
	No	54	22	1	

## Discussion

In this study, the sociodemographic of the participants’ sex was different from other studies: our study consisted of 56% men and 44% women. The percentage of women was higher in the study carried out in Dangla (men = 48.3%, women = 51.7%) (8). This might be due to a high level of participation in social activities or the suppression of women in that study area. In the study carried out in Gondar Hospital, 54% of participants were men and 46% were women (20); in a study carried out in Istanbul, Turkey, 30.8% were women and 69.2% were men (21). However, many studies show that men had more suicidal ideation and attempts than women.

In our study, the primary and secondary education level had 5.86 times and 3.61 times more likely risk of association of suicidal ideation and attempts (AOR = 5.86, 95% CI 1.30–26.41; and AOR = 3.61, 95% CI 1.01–12.88, respectively). The level of education in the present study was higher than that in the study carried out in Amanuel Hospital (AOR = 1.63, 95% CI 0.68–3.89; and AOR = 2.51, 95% CI 1.11–5.68, respectively) (22). This might be due to academic failure or low satisfaction in the future of their job due to their education.

In addition, hopelessness was a more likely risk of association with suicidal ideation and attempts. The present study had a 2.79 times more likely risk of association of suicidal ideation and attempts (AOR = 2.79, 95% CI 1.11–6.98). This was slightly higher than that in a study carried out in Amanuel Hospital (AOR = 2.510, 95% CI 1.459–4.320) (22). This might be due to those psychotic patients who do not see futurity, have no plans to live, do not think positively, and are not anxious to take any opportunities that might put them in a better position.

In our study, we determined that suicidal behavior was significantly less common in patients with mental illness who were divorced compared with those who were single or never married. This study was also lower than that in a study carried out in the USA (AOR = 2.1, 95 CI 1.83–2.33) (23). Thus, studies further suggested that the highest risk increase was in never-married individuals and those who were separated or divorced (24). Similarly, being single was associated with an increased risk of suicide in women aged 20–34 years (20). Furthermore, individuals who had poor social support were 2.25 times more likely to have suicidal ideation and attempts than patients who had strong social support. This was similar to the results from recent studies (25, 26).

In this study, patients with a family history of mental disorders significantly increased the rate of suicidal ideation and attempt, which is 1.94 times more likely risk of suicidal ideation and attempts (AOR = 1.94, 95% CI 1.12–3.36), which is slightly lower than or comparable to a study carried out on psychiatry patients at Jimma University Hospital (AOR = 2.25, 95% CI 1.11–4.57) (2). In addition, suicidal behavior was more likely to occur in patients with a family history of suicidal behavior when compared with patients without a family history of suicidal behavior. Our findings seemed to be similar to those in studies showing that a history of suicide in the family could increase the risk of suicidal behavior in patients with mental illness (21, 27). In their study, Trémeau et al. indicated that a family history of suicide significantly increased the risk of suicide attempts, with higher lethality and frequency of suicide attempts (28).

As a limitation, institutional-based studies could not address those adolescents outside the institution, which assessed the lifetime prevalence of suicidal ideation and attempts at the point of current prevalence during the stated period. In addition, this study assessed their status of suicidal ideation and attempt based on their stated response; in this case, individuals may not disclose their actual thoughts or attempts about suicide.

## Conclusion

In our study on the sociodemographic of sex, men were more affected by suicidal ideation and attempts than women. The prevalence of suicide ideation and attempts was high, showing a significant public health issue among hospital-attending and chronic mentally ill patients that requires a great emphasis. These risk factors were primary and secondary grade/school education, feelings of hopelessness, stopping mental illness medication, family history of mental illness, family history of suicide, being divorced, social support of the patient, and current psychotic diagnosis of other diagnosis of illness had positively or more likely risk of association with suicidal ideation and attempts.

Therefore, suicide prevention needs to involve different actors and disciplines working at suicide non-governmental and non-profit organizations, universities, and civil society at different levels. Early detection and intervention are the single most important prevention strategy of suicide in patients with mental illness. Thus, clinicians are strongly recommended to identify those patients with the abovementioned high-risk factors. The prediction of suicide in patients with mental illness is complex and difficult, and efforts at prevention should also focus on optimizing social support and adherence to psychiatric medication for people with mental illness. Furthermore, St. Paul’s Hospital should have a different psychiatry treatment center and rehabilitation center that separates them from other services.

## Data Availability

The data analyzed in this study is subject to the following licenses/restrictions: The data will be available based on reasonable request to the author. Requests to access these datasets should be directed to Awol Dawud, awol.mod@gmail.com.
